# Working Women in Large Towns

**Published:** 1890-06-28

**Authors:** 


### Working Women in Large Towns.

### V.—WOMEN AS TAILORS.

The next group of working women that claims our notice is the tailoresses, much of whose work comes under the head of what are known as the "sweated industries." And as we shall have occasion to speak of several of these industries (indeed, we have already considered one—Boot and Shoe making), both now and when considering "home work," it may be as well to devote a few words to describing the nature of sweating before we proceed further.

By a "sweated industry" we mean one upon which the terrible force of unrestrained competition has imposed certain conditions—long and irregular hours, miserable pay, overcrowded and unhealthy workshops. Before answering the question, "What is a sweater?" we would remark that Miss Beatrice Potter speaks of the sweater as having at least a threefold personality, one of this trinity being (let us mark it well) the "ignorant consumer." But in common parlance, the sweater is the man who takes work from the large manufacturers (either with or without the intervention of a "middleman"), and gives it out to a small band of workers, who will either take the work home, or do it in the sweater's workshop. This sweater has had many hard words said of him; and as Mr. Booth remarks, popular opinion has decided that the larger sweating master is a monster of inhumanity. But although there are doubtless many hard and mean men to be found in the sweating line —as in every other, the real truth seems to be that it is the whole sweating system, rather than the sweater himself, which is really responsible for the untold miseries of those who work in such trades. The small sweater is generally ground down himself, and could not give higher wages if he would; he works as hard and as long as any of his *employés*, and very often earns less than the most skilled hands. Again, it is indisputable that the workers for the larger kind of sweater are, as a rule, better off than are those who work for the smaller; so that we must relinquish, once and for all, the hope which we would fain cherish, that if that monster "prince of the sweating system" could only be got hold of, and made to disgorge his profits, the problem would be solved. We must not, however, linger longer over this fascinating, though painful subject, but pass on to consider the case of the tailoresses.

It is probable that there is a larger range in the earnings of English tailoresses than in those of any other class—save and except, perhaps, needlewomen. None of them can make a garment out and out—except, indeed, the miserable women at the very bottom of the coat trade, who will take work at prices which would be scornfully refused by the smallest sweater, and make the coats throughout for sevenpence each. Why it is we know not; but women's work is always lacking in "form," and thus the most difficult part of the tailoring is never allowed to pass out of the hands of men, even in the best West-end work-rooms. Still, the West-end tailoresses earn good money, some taking as much as thirty-six shillings a-week. On the other hand, their work-rooms are too often small and stuffy, and they feel much alarm lest they should be gradually displaced by the foreign (male) labour, which has already begun to invade their trade. Again, some East London trouser-makers will sometimes clear five shillings a-day, either as machinists or "finishers." But it is in the lower sections of the tailoring trade that the great majority of female workers are to be found. Large numbers of them work at home; but those whom we have now to consider sit all day in crowded workrooms, felling seams, making button-holes, and generally "finishing" pair after pair of "slop" trousers, or, it may be, vests. In some of the work-rooms the staff consists of six, eight, or ten men, and one girl. We give a short description, from *Toilers in London*, of one of the worst of these sweating dens, though it is to be feared that there are very many quite as bad:—

"The room, twelve feet square, held ten people—the sweater, eight men and one girl. The window panes were nearly all broken, and filled up with paper or canvas. The gas was burning, also a large coke fire. There was no ventilation whatsoever; and the men were covered with sweat, though they had on the veriest apology for clothes. The girl wore an old ragged dress, open at the chest. Her forehead was studded with perspiration, and her hair was wet. . . . All the men were Jews, but the girl was a Gentile."

In other work-rooms the staff comprises but two men—pressers—and a motley assemblage of women and girls, perhaps thirty in number, who work by the piece at various kinds of trousers, known as "threepence ha'pennies," "fourpence ha'pennies," and so forth, as skill permits. A graphic picture of such a work-room was given by Miss Potter in the *Nineteenth Century* for September, 1888, entitled "A Work-Girl's Diary." As to hours, all these workers are nominally under the Factory Acts, and the law could not probably be systematically violated in workshops of this size; though the women are "turned upstairs" now and again when there is a press of work, and it is more than suspected that in this, and other industries, they will sometimes come at six instead of eight, thus making up an extra two hours without fear of being overhauled by the inspector, who, poor man! can hardly be expected to be up and abroad at that unearthly hour. But how is the inspector to control the numberless sweating-dens which are springing up like mushrooms in the Jews' quarters of East London? He does not even know of their existence, for they are in basements, garrets, back-yards, wash-houses, and all sorts of unsuspected places; sometimes they are merely the living rooms of the sweater and his family, and if the inspector pays a sudden visit, the women will be hurried off into a bed-room, whither he has no right to follow.

The wages, as has been said, vary so greatly, according to the skill and efficiency of the worker, that it is difficult to give much information as to their general scale; but there seems to be no doubt that the great majority of tailoresses are "unskilled hands." Leaving out of consideration the home-workers, whose pay is indescribably miserable, we may say that many thousands of women are working long hours for a daily wage ranging from two-and-threepence to eighteenpence, a shilling, and even less. Add to this the fact that there are always slack seasons in the trade, and that, as Miss Potter calculates, the average work per week in the tailoring trade is "three days for medium shops and average labour, and two and a-half days or under for the great majority of permanently unskilled workers"; and it becomes evident that we are face to face with an amount of misery which cannot well be over-estimated.

We must not omit to mention that, while the bulk of the tailoring trade falls to the share of Londoners, there are various provincial factories where cheap clothing is made, which are said to press the London trade hard, owing probably to the superior cheapness of living in the country. In Leeds, again, there is a very large tailoring community, which appears to be rapidly increasing. Most of the men employed in it are Jews, but they are assisted by large numbers of Gentile women; and although the Labour Correspondent of the Board of Trade pronounces Leeds tailors and tailoresses to be, as a whole, better off than their London brethren, this by no means proves, alas! the absence of distress.

Efforts have lately been made, and not without success, to form unions among the tailoresses both of London and Leeds; and, certainly, if any workers need the protection which combination affords, it is these poor women.

